# Retroversion of the Uterus

**Published:** 1886-11

**Authors:** 


### *RETROVERSION OF THE UTERUS.*

From a Clinical Lecture by PROF. PARVIN. Reported in the *N. C. Medical Journal.*

The second patient that I have to show you is a woman aged forty-nine, who has had three children, the last one six years ago; she has had no miscarriages. When I asked her about her history, she says that she has never been well since her first confinement. The second child was born one and a half years after the first, and the third followed the second in one year and five months. So you see she has had three pregnancies, following each other in rapid succession, and she tells us that she has nursed all of her children. She was thirty-two years old when her first child was born, and this fact causes me to speak of her as having been an old primapara. I believe that it is customary in fashionable society never to call a lady old as long as she is a *miss*, but this rule does not hold good in obstetrics, for at thirty we would say that a woman was an old primapara. Now, in connection to this fact, I want to call your attention to the circumstance that the duration of her first labor was only seven hours, whereas we are accustomed to say that the duration of labor in a primapara is usually twelve hours. She tells us that her chief complaint is pain in the back and in both sides, but most marked on the right. Now, I ask her if any position she may assume will afford relief to this pain in the back, and I want you to listen carefully to her answer, and note it well, for there is something very significant in her reply. She says that when lying down she will frequently stuff a pillow into the small of her back, and that this affords relief. I asked her if any one told her about this proceedure, and she answers no, that she just did it of "her own accord." Well, now, you know that instinct will cause a dog to eat grass, under certain circumstances, and so also will instinct often direct the movements of the higher animals, and direct them so correctly that we can often derive a deal of information from these instinctive acts. From this instinctive effort to afford some artificial support to the back, we must needs infer that there is some extreme pressure on the back, and so, in truth, there is, for it is usually in cases of retro-flexion where the uterus is pressing down on the sacrum that we find the women stuffing pillows under their backs. This woman tells us that her menstrual periods are regular, but that she has some discharge, some leucorrhœa, or, as she tersely puts it, the "whites." By-the-way, never ask a patient, even among your most intelligent ones, if she has "leucorrhœa," for if you do she will not understand you, she will think you are talking Sanscrit, or some other dead language, and may possibly give you a misleading reply rather than admit her ignorance of the nature of your query. On the other hand all women know what you mean when you ask them if they have the "whites," and I would therefore advise you to confine yourself to the more homely, but more universally understood term. I find that this case has been put down on the register as one of laceration of the cervix. Well, in a measure, this is correct, there is, no doubt, a laceration, but there must also be some further trouble behind and beside this to account for the symptoms she presents. She has a copious discharge, which really means that there is a uterine catarrh, and I would here say that I think it is best to call all non-sanguinolent discharges from the uterus uterine catarrh. This is simply a hyper-secretion. I remember years ago, when I was passing through my examination for the degree of M. D. (and I suppose that at this time of the year many of you have peculiar interest in these examinations), one of the professors asked me as to the effect of irritation on secretions. When the irritation is but slight in degree it merely produces an increase of secretion, while when it is more severe it not only causes this increase, but it also produces a perversion of the secretion. In this case the discharge is like the white of egg, from which we infer that it comes from the uterus, for if it was from the vagina it would be more milky in character. A woman with uterine catarrh does not usually have a vaginal leucorrhœa, but let her walk for several squares, and she will find her garments soiled with a discharge that has come from the vagina and has been caused by the irritation of walking. Now, at times this discharge is yellowish and contains pus, which occurrs when the grade of irritation is temporarily higher, and, as I have said, as a consequence, the character of the secretion is perverted. The laceration will not account for all the symptoms, and the fact that there is uterine catarrh indicates to us that there is inflammation, or at least congestion of the uterus. If this discharge were thin and watery, then I would say that it came from the cavity of the uterus, but, as it is thick and sticky, we know that it comes from the cervical canal. A still further test of the origin of the discharge could be made up by the use of litmus paper, if it was found to be alkaline, then we should know for sure that it came from the cervix. Well, now, she further complains of this pain, which Dr. Matthews Duncan has so aptly called *Sacralgia*, not a very euphonious, but at the same time a very expressive word. It is a girdle of pain, as it were, starting in the small of the back and passing nearly around the body. Now, this pain means that there is most probably a posterior displacement of the uterus. You know that the utero-sacral ligaments play a very important part in keeping the uterus in its normal position, and while these ligaments maintain their integrity the uterus cannot become displaced, but when, through their relaxation, the fundus of the uterus falls backwards and the cervix is in consequence tilted forwards, there must be, as you can readily comprehend, a stretching, a tension of these that will cause this pain. But in addition to this sacralgia, there is also pain in front, in the right and the left. How can we account for this? Very readily, by supposing that this uterus is dragging on the round and broad ligaments. When a patient comes to you complaining of such pain you can rest pretty well assured that you have to do with either a prolapsed or a retroverted uterus. Well, then, we have satisfied ourselves that this woman has retroversion. Now, this condition might be overlooked in the laceration. She might go to a doctor, who, discovering the laceration, and supposing it to be the cause of all the trouble, would operate on it. This operation might have the effect of lessening the catarrh, and it would most likely do so, owing to the combined influence of the rest which the operation would entail and the depletion which the escape of blood during the operation would entail, thus reducing the congestion. But in a short time the catarrah would be as bad as ever, because the operation would not radically remove the cause. It will sometimes happen that an operation for laceration of the cervix will cure a retroversion, and that the uterus will hereafter maintain its normal position. This result I would ascribe to the setting up of an inflammation in the utero-sacral ligaments, as a consequence of which they contract, thus drawing the cervix backwards and tilting the fundus forwards. But while this does occasionally occur, yet I would advise you not to count on it, for if you do you will be frequently disappointed. It is not enough merely to operate on the laceration; in fact, I doubt very much whether such an operation would be advisable in this case, where the laceration seems to be producing comparatively so little inconvenience. We will place the woman on her left side and draw the uterus downwards, then draw the cervix backwards and endeavor to keep it in position by a pessary. We will also resort to injections of hot water to reduce the congestion, paint the cervix with Churchill's iodine, and use tampons of glycerine. If the catarrh persists, then we will operate. I speak as I have about the operation for lacerated cervix to put you on your guard, for there are some who seem to think that this operation is a sure cure for all the ills that woman flesh is heir to. Baker-Brown, of England, who was, in his day, one of the greatest of gynæcologists grew gradually to believe that all the troubles of women, of a nervous nature, were due to the clitoris, and he commenced a crusade against this organ. He would preserve the clitoris from all his patients, young or old, married or single, until he had as great a collection of these little organs as the butcher has of similar organs from cattle; he became wild on the subject. As a result he was ignominously expelled from the Obstetrical Society of London, and died a paralytic, poor and in seclusion. Such cases should warn us against hobby-riding, against allowing any theory, however plausible it may seem, to absorb or reason against considering any operation a panacea. Baker-Brown was disgraced by his one-idea views. I fear that the profession are tending in the same direction now with reference to another organ of the female economy, that is being used to account for all variety of nervous disorders. I have recently read a work wherein the author says that it would seem as though the profession was rapidly growing to regard laparotomy as the panacea of gynæcology, and he says that he believes the day is not far distant when the removal of a sound ovary for the cure of hysteria, epilepsy, insanity and allied diseases will be considered as unjustifiable and as criminal as is clitoridectomy.

